# A potential gateway to understanding liver disease development: peripartum lipid fluctuations in dairy cows

**DOI:** 10.3389/fcell.2024.1370717

**Published:** 2024-11-26

**Authors:** Laura Vogel, Markus Güttler, Kirsten B. Theinert, Teja Snedec, Kristin Reichelt, Fabian Pietsch, Melanie Schären-Bannert, Fanny Rachidi, Gabriele Dobeleit, Herbert Fuhrmann, Joachim Spilke, Frank Edlich, Alexander Starke

**Affiliations:** ^1^ Clinic for Ruminants and Swine, Faculty of Veterinary Medicine, University of Leipzig, Leipzig, Germany; ^2^ Institute of Biochemistry, Faculty of Veterinary Medicine, University of Leipzig, Leipzig, Germany; ^3^ Biometrics and Informatics in Agriculture Group, Institute of Agriculture and Nutrition, Martin-Luther-University, Halle, Germany

**Keywords:** lipid metabolism, liver disease, NASH, NAFLD, peripartum period

## Abstract

Current lifestyles are leading to a worldwide increase in metabolic liver diseases that favor the development of liver disease. Changes in hepatocytes are caused by altered lipid concentrations, oxidative stress or toxicity by individual lipids. The complexity of the underlying processes and differences of the pathology to proposed rodent models makes the development of an effective targeted therapy difficult. The lipid mobilization that occurs in dairy cows in the *postpartum* period could be a natural model for the metabolic stress commonly observed in the development of liver diseases. We therefore used gas chromatography and histopathological staining techniques to analyze lipid patterns in diparous and multiparous cows during the peripartum period. The most striking change in lipid composition is the homogenous increase in palmitoleic acid (C16:1n7) content in all cows around the time of calving, with multiparous cows exhibiting consistently higher C16:1n7 levels by the end of the study. Elevated C16:1n7 levels have a potential key role in the development of non-alcoholic steatohepatitis (NASH) and tumorigenesis in the liver. Changes in C16:1n7, therefore, support the idea that lipid mobilization in dairy cows could serve as model for various liver diseases, such as nonalcoholic fatty liver disease (NAFLD) or NASH development.

## 1 Introduction

The primary etiological risk factors for chronic liver inflammation are a spectrum of infectious origins, such as chronic hepatitis B (HBV) and hepatitis C (HCV), as well as pathological alcohol consumption, exposure to dietary toxins and metabolic liver diseases (Xiao et al., 2019). Advancements in healthcare have led to a decline in chronic HBV and HCV prevalence in the Western world. Conversely, a rising incidence of metabolic liver diseases, induced by excess body weight and diabetes, resulting in nonalcoholic fatty liver disease (NAFLD), non-alcoholic steatohepatitis (NASH) and even tumorigenesis in liver (Sung et al., 2021). Excessive accumulation of visceral fat due to hypernutrition can lead to insulin resistance, a prominent factor contributing to the buildup of fat in liver tissues (Lo Iacono et al., 2007), which is characteristic for NAFLD and its more inflammatory counterpart, NASH (Ohki et al., 2009). Subsequent processes triggered by an augmented total lipid yield, such as oxidative stress, and the specific direct toxic effects of certain free fatty acids (FFA) or other lipids on the liver, can induce alterations in hepatocytes (Neuschwander-Tetri and Caldwell, 2003; Cusi, 2012).

The overall effects of these changes, however, are not only determined by individual lipid types or metabolic pathways (Alkhouri et al., 2009). Rather, numerous complex mechanisms are involved within the hepatic lipid metabolism, which research is only beginning to understand. The inherent intricacy of the underlying factors complicates the development of an effective targeted therapy (Paul et al., 2022). One reason for the lack of understanding the metabolic processes governing the development of pathology is the limited access to human material at crucial NAFLD stages of development and the fact that isolated cells do not adequately reflect the living organism. Conversely, established rodent models primarily rely on transgenic animals or pharmacological/chemical/dietary inducers to create NAFLD/NASH. However, these methods often present major commitment, and limited comparability to human conditions (Zhong et al., 2020). Research needs for a natural model of NAFLD/NASH in mammals are substantial.

A common process in the dairy cow could meet those needs. Dairy cows in the beginning of lactation (*postpartum* period) undergo energy deficiency and mobilize lipids from storage fat (Drackley, 1999). Peripheral storage lipids are accumulated in the liver tissue and reach a maximum capacity for lipid metabolism leading to pathological NAFLD. Lipids that can neither be metabolized nor transported by additional synthesis of apo-lipoproteins require storage in the liver (Mensenkamp et al., 1999). Multiparous cows show higher total lipid and triacylglyceride concentrations in liver tissue during the *postpartum* period (Janovick et al., 2011). The increased triglyceride concentration appears to be associated with an increased risk of NAFLD (Reid, 1980; Grummer et al., 2004; Janovick et al., 2011). Associated inflammatory processes could also trigger liver fibrosis (Zhang et al., 2023). While general changes in fat content may play a role in NAFLD, specific lipids are likely to govern the central processes. Therefore, our research focused on analyzing the lipid patterns of diparous and multiparous cows in the *postpartum* period in order to identify key lipids within the framework of liver metabolism that could provide clues for therapeutic strategies for NAFLD/NASH.

## 2 Materials and methods

### 2.1 Animals, husbandry and experimental design

The study was conducted in a commercial dairy farm with 660 German Holstein cows in Saxony. The rolling average milk production was 10,747 kg with 3.73% fat and 3.33% protein, and cows were housed in a free-stall and fed a total mixed ration (TMR) as described (Schären et al., 2021). A detailed description of the study setup and husbandry routines (e.g., feeding, grouping) are described by Schären et al. (2021). Briefly, inclusion criteria at 14 days antepartum (a.p.) calculated from the expected calving date were: ≥2nd lactation, pregnant, clinically healthy, no treatment 28 days prior to first sampling or persisting withdrawal period (exception: dry-off treatment), and expected dry period longer than 70 days. For this trial, twelve clinically healthy cows in their 2nd to 6th lactation were recruited. Lactation 2 cows (DP): age, 3.4 years [3.0–3.8 years, mean (min-max)]; BCS (Body Condition Score) 14 days a.p., 2.5 [1.8–3.5; according to Edmonson et al. (1989)]; height at withers, 143 cm (139–146 cm); BW, 657 kg [536–766 kg; weighed using a Texas Trading Squeeze Chute (Version S 04 with weighing scale)]; and 305-day milk yield in previous lactation, 9,603 kg (7,685–11,818 kg, Supplementary Table 1). For lactation 3 or greater cows (MP): age, 5.2 years (4.0–7.5 years); number of lactations, 3.8 (3.0–6.0); BCS 14 days a.p., 2.4 (1.8–3.3); height at withers, 148 cm (143–152 cm); BW, 682 kg (594–794 kg); 305-day milk yield in previous lactation, 11,802 kg (10,147–14,470 kg).

### 2.2 Clinical and laboratory monitoring

The cows were monitored clinically from 14 days a.p. until the end of the study period 42 days *postpartum* (p.p.). The clinical findings were transferred to a clinical scoring system for different health traits as described (Schären et al., 2021). Blood samples were collected from a jugular vein into plastic tubes without anticoagulant for the determination of the non-esterified fatty acids (NEFA) concentration in serum, and into plastic tubes containing lithium heparin for the measurement of plasma concentrations of albumin, ß-hydroxybutyrate (BHB), bilirubin, creatinine, cholesterol, glucose, lactate, urea, triacylglycerol, calcium, chloride, potassium, magnesium, sodium, phosphorus, and total protein, and the activities of alkaline phosphatase, alanine aminotransferase (ALT), aspartate aminotransferase, creatine kinase, gamma glutamyltransferase (GGT), and glutamate dehydrogenase (GLDH). The chloride concentration of voided urine was determined. All laboratory results were within the reference intervals.

### 2.3 Liver tissue sampling

Liver biopsy (1–2 g liver fresh weight) was done at 14 days a.p. and 7, 28, and 42 days p.p. under local anesthesia [Isocain ad. us.vet. 20 mg/mL; Selectavet, 20 mg/mL procaine hydrochloride (corresponding to 17.3 mg/mL procaine and 0.025 mg/mL epinephrine, Weyarn/Holzolling, Germany)] as previously described (Gohlke et al., 2013). The biopsy samples were stored for about 1 week in liquid nitrogen and then at −80°C until processing. Difficulty in predicting parturition, variable farm management schedules, and high operator workload resulted in minor deviations in the actual from the scheduled sampling days:14 [11, 13–6; scheduled sampling day (actual sampling day: mean, earliest-latest)] d a.p.; 7 (8, 6–9) d p.p., 28 days (28, 26–29) d p.p., and 42 (42, 40–44) d p.p.

### 2.4 Histopathologic assessment

The evaluation was performed as previously described by Pietsch et al. (2021). Briefly, liver samples were histologically assessed for various characteristics, including fatty infiltration (severity of fat deposition and size of lipid droplets), glycogen storage (extent of glycogen accumulation and particle size), as well as degenerative, inflammatory, fibrotic, and proliferative changes, such as liver cell degeneration and the extent of perisinusoidal fibrosis. The evaluation was performed “blindly” by a trained veterinarian using a light microscope (CETI-AC 22V IN, TOPIC-B) with magnifications ranging from ×40 to ×400. Five liver lobules were randomly selected and evaluated in each sample using ×10, ×20, and ×40 objectives. Histological alterations were classified as focal (observed in a single liver lobule), multifocal (observed in two or more liver lobules), or diffuse (observed in all five liver lobules).

A standardized scoring system was applied to assess individual histological changes (Pietsch et al., 2021). The evaluations were based on both qualitative and quantitative criteria. The size of lipid droplets was categorized as small (droplets red to orange, smaller than the hepatocyte nucleus), medium-sized (droplets similar in size to the hepatocyte nucleus), or large (droplets larger than the hepatocyte nucleus). Regressive nuclear changes were defined by degenerated cell nuclei, where the nucleus (blue) was displaced peripherally by a fat vacuole, with evidence of nuclear death.

The degree of fatty infiltration was defined accordingly to Pietsch et al. (2021). The total score for each of the three zones was calculated and summed [Σ (size of lipid droplets × severity of lipid infiltration of hepatocytes)_c,i,pl_, where c = centrilobular zone, i = intermediate zone, and pl = peripheral or periportal zones]. The severity of lipid infiltration was determined from the average score across the five liver lobules and categorized as no lipid infiltration (0), mild (1–6 points), moderate (7–15 points), or severe (16–27 points). The size of the lipid droplets was compared to the size of the hepatocyte nuclei in Sudan III-hematoxylin (SIII; Riedelsheimer and Büchl-Zimmermann, 2015) -stained sections, with the predominant droplet size used for analysis.

### 2.5 Plasma and liver lipid extraction

Sample Preparation: Liver samples (∼0.5 g) were homogenized using an ultraturrax in a diluting agent comprising chloroform and methanol (v:v = 1:1) at a ratio of 9 mL per 1 g of tissue. For subsequent processing, 1 mL of homogenized material was diluted in a solution consisting of 0.8 mL double distilled water, 0.5 mL chloroform, 1.0 mL methanol, and 0.5 mL methanol with 0.2% (1 µg) butylated hydroxytoluene (BHT).

Following vortexing and incubation for 10 min, phase separation was achieved using 1 mL chloroform and 1 mL double distilled water, followed by centrifugation at 3,112 g for 10 min at 15°C. The lower phase was isolated for further analysis. The lipids were diluted in chloroform:methanol (v:v = 1:1) to achieve a concentration of 10 mg lipid/mL.

By preparative thin-layer chromatography on 0.5 mm silica PSC plates (5 cm × 5 cm, Merck, Darmstadt, Germany) the different lipid fractions were separated. Subsequently, 1.5 mL chloroform:methanol (v:v = 1:1) was added twice, followed by incubation for 1 min and centrifugation at 15,000 U/min for 3 min.

### 2.6 Sample preparation for gas chromatography (GC)

Lipids were subjected to methanolic hydrochloric acid (500 μL) for esterification, followed by extraction with n-hexane (250 μL) and the addition of 500 μL internal standard (IS) (Seidel et al., 2005). The IS used consisted of 0.08 mg L-α-phosphatidylcholine-C17:0 dissolved in 1 mL methanol with 0.2% BHT as an antioxidant. Esterification took place for 30 min in a water bath at 85°C. After cooling, an additional 500 μL n-hexane and 1 mL distilled water were added for phase separation. The samples were vortexed for 1 min and then centrifuged at 3,112 g for 10 min at 4°C. Approximately 500 μL of the resulting upper hexane phase was extracted and dried under nitrogen. The fatty acid methyl esters (FAME) were subsequently reconstituted with 70 μL n-hexane. Measurement tubes were stored at −25°C until analysis.

### 2.7 Gas chromatography

The measurement and quantification of FAME were performed using a Varian CP 3800 (Agilent, Waldbronn, Germany) gas chromatograph with Omegawax TM 320 (30 m × 0.32 mm diameter × 0.25 μm film, Supelco, Bellefonte, PA, United States) column after 1 μL sample “sandwich” injection at 250°C with initial split ratio: 1:50, after 1 min 1:100, after 20,30 min 1:3. Carrier gas was Helium (1.5 mL/min). Recording and evaluation of the Flame Ionization Detector (FID), 250°C, chromatograms were conducted using the Star 6.0 software (Agilent), employing the IS as a reference peak. For the quantification of individual fatty acids, standard mixtures (FIM-FAME-6 mix, Matreya LLC; C20:5n3 FAME, Supelco; C22:5n3 FAME, Supelco; CLA, Supleco; C13-C18 iso- and anteiso-BCFA-ME, Larodan Fine Chemicals) were used. Further identification of unknown peaks was performed using the OmegaWax Column Test Mix, (Supelco), PUFA-1 Cod Liver Oil (Supelco) and PUFA-2 Animal Source (Supelco).

The obtained µg of fatty acid methyl esters was calculated into fatty acids and normalized to their reference size.

### 2.8 Data and statistical analysis

#### 2.8.1 General remarks

All analyses were done using SAS 9.4 (SAS Institute 2016, Cary, NC, United States; proc univariate, proc mixed, proc glimmix, proc iml).

A linear mixed model was used for all variables because they involved repeated measures. Degree of freedom approximation based on Kenward and Roger (1997) was required for the F-test and the analysis of differences and confidence intervals of the LSM in the original or transformation scales as well as the OR in case of the threshold model.

Differences between LSM were analyzed using the Tukey-test. In the case of significant interactions, the comparison of the LSM refers to the factor combination groups*sampling days. However, only comparisons with biological importance were carried out. Therefore, the possible comparisons of groups and sampling days (28 comparisons in total) were limited to comparisons of sampling days within groups (6 comparisons each) and to comparisons of groups within sampling days (one comparison in total). Accordingly, F- and Tukey-tests of interactions are given only for these comparisons.

#### 2.8.2 Percentages of individual fatty acid content in the hepatic lipid fractions and plasma, concentrations of ALT, AST, GGT, GLDH, and BHB in blood

Group (Lact2 and Lact≥3) and sampling days and interactions between these variables were fixed qualitative effects. Repeated measures within cows were taken into account by choosing an appropriate covariance structure [ar (1), toep (2), cs, un, vc] with the help of the Akaike information criterion with correction for finite samples (AICC).

Deviations from the scheduled sampling days were considered as an additional independent variable via a linear covariate. Additionally, estimation of milk yield on d 42 p.p. was done (see section milk) and used as covariable as well as degree of fatty liver infiltration, total severity of fibrosis, and severity of hepatitis. The covariables were included in the evaluation model if the associated regression coefficient were significant. For absolute concentrations, log(e) and square root transformations were tested, and for percentages of lipid fractions, arcsine square root transformation and log(e) transformations were tested. Type of transformation was chosen based on the best fit to the normal distribution according to the Shapiro-Wilk test. For traits that required transformation, the least square means (LSM) given in our results represent back transformations.

#### 2.8.3 Histopathologic scores

For analysis of the ordinal variable histopathologic score with repeated measurements within cows, the cumulative logit (random effect) model based on the threshold concept was used (McCulloch and Searle, 2000).

Let *Y*_*ijm*_ be the ordinal response (with r categories) of animal *m* in sampling day *i*, and lactation class *j*. Further, let *Z*_*ijm*_ be the underlying continuous latent variable and *θ*_*n*_ cutpoints (thresholds with n = 1, r − 1). Then, at given animal effect, the cumulative logit random effect model is given by:

$$\text{logit}\left[P\left.\left(Y_{ijm}\leq n\right)\right]=\text{logit}\left[P\right.\left(Z_{ijm}\leq \theta_{n}\right)\right]=\theta_{n}-\eta_{ijm}$$

with $\eta_{ijm}=\mu +\alpha_{i}+\beta_{j\;}+b_{1}\cdot x_{ijm}+{\;a}_{m}$

where $\alpha_{i}$ fixed lactation class effect (fix; l = 1, 2) and $\beta_{j}$ fixed sampling day effect (*j* = 1,2,3,4, where 1 = 14 days AP, 2 = 7 days PP, 3 = 28 and 42 days PP) and ${\;a}_{m}$ random effect of animal m ( $a_{m}∼N(0,\sigma_{a}^{2}$ ). The difference between effective and planned sampling time point (x_*ijm*_) is accounted for by the linear regression coefficient b_1_.

Because of the existing model complexity attributable to the main effects, we did not examine interactions when considering the sample size.

The implementation provides F-test (related to the effects in the linear predictor), cumulative probabilities, and odds ratio (OR).

#### 2.8.4 Statistical evaluation of the OR

Odds describe a probability ratio in the sense that the ratio of a probability of occurrence to the opposite probability is formed: Odds = P(Y = 1)/(1-P(Y = 1)). For statistical evaluation of the OR, simultaneous confidence intervals were provided using the Bonferroni correction.

The OR and 95% confidence intervals (CI) were calculated to describe differences among different levels of the effects sampling day, and lactation number. The OR was considered to be significantly different from the value one when their CI did not overlap with the value one.

## 3 Results

### 3.1 Omega-3 and omega-6 fatty acids increased in the liver fat of diparous cows

**FIGURE 1:**
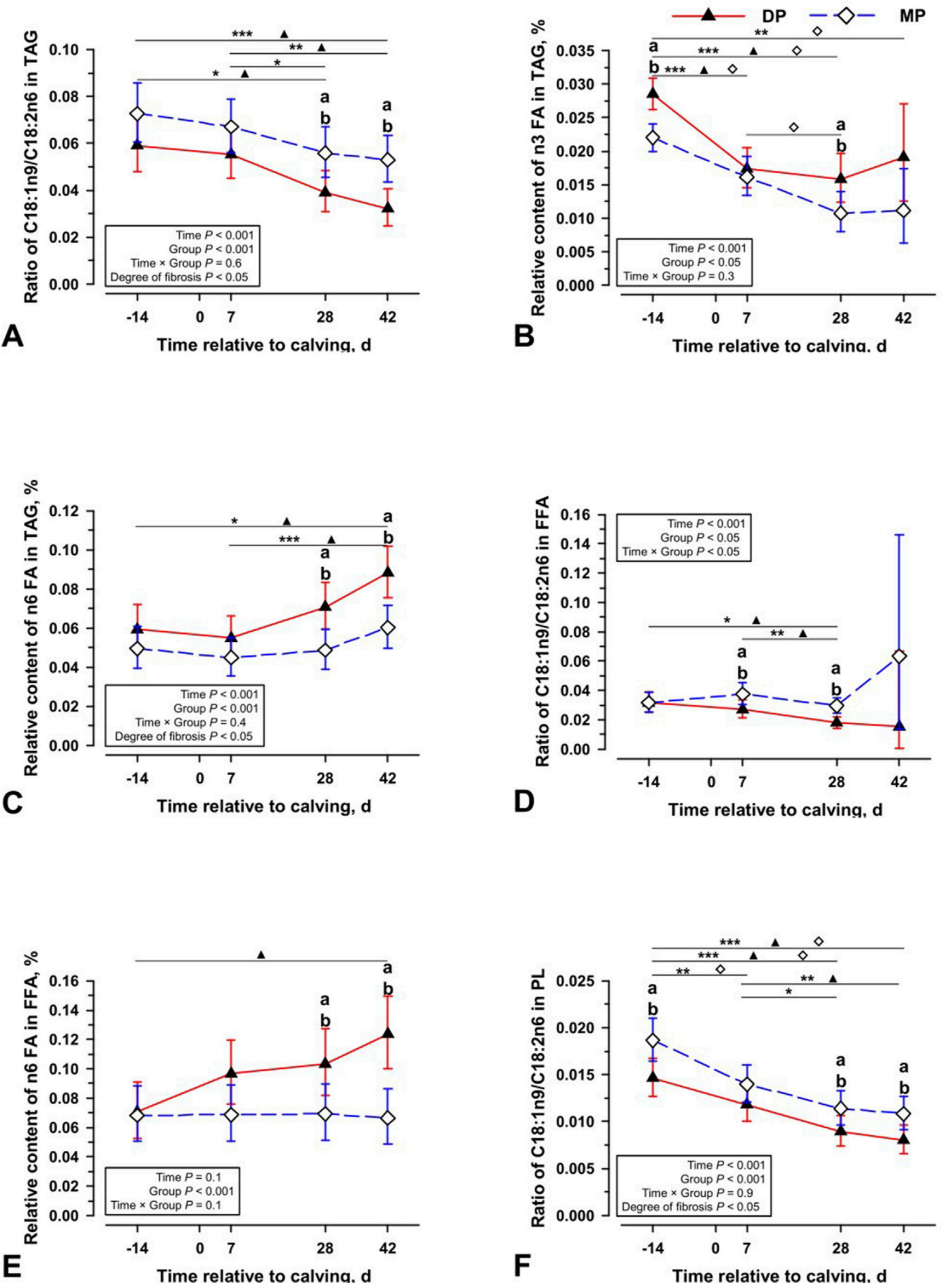
The ratio of C18:1n9/C18:2n6 in triacylglycerol [TAG; **(A)**], relative content of n3 fatty acids (FA) in TAG **(B)**, relative content of n6 FA in TAG **(C)**, ratio of C18:1n9/C18:2n6 in free fatty acids [FFA; **(D)**], relative content of n6 FA in FFA **(E)**, and ratio of C18:1n9/C18:2n6 in phospholipids [PL; **(F)**] at four periparturient sampling days [−14 = d 14 antepartum (a.p.); 0 = parturition; 7, 28, 42 = d 7, d 28, d 42 *postpartum* (p.p.)] in German Holstein cows with different age (two groups; DP = lactation 2, n = 6; MP = lactation 3 or greater, n = 6) as Least Square Means (LSM) and their 95% confidence intervals (error bars). Differences between the time of sampling are marked with asterisks (*p < 0.05; **p < 0.01; ***p < 0.001), differences between time of sampling within the groups marked with symbols (▲ DP; ⍚ MP) and differences among the lactation groups at the respective time point marked with different lowercase letters: (a, b) indicate significant differences (*p* < 0.05).

To begin our analysis, we tested whether a difference exists in the proportion of polyunsaturated fatty acids (PUFA) in the storage liver fat between diparous and multiparous cows. It should be noted that changes were observed in the total liver lipid fractions of triglycerides and cholesterol esters (Supplementary Table 2). A difference between diparous and multiparous cows is evident in the liver fat content changes, with increasing fractions of triglycerides in particular in diparous cows up to day 7. Only in multiparous cows is a further increase in the lipid content of both triglycerides and cholesterol esters observed up to day 28, which only then decreases again. The ratio of oleic acid (C18:1n9) to linoleic acid (C18:2n6) shows that diparous cows contain significantly higher proportions of polyunsaturated fatty acids in their liver tissue triacylglycerides (Figure 1A). Especially towards the end of the observation period, these animals show a tendency towards a higher content of omega-3 fatty acids and a significantly higher proportion of omega-6 fatty acids in the liver tissue storage fat (Figures 1B, C). There is also a significantly higher proportion of polyunsaturated fatty acids in the free fatty acids and thus the ratio between oleic acid and linoleic acid is also lower in young cows (Figures 1D, E). The higher proportion of omega −3 and omega −6 fatty acids is not reflected in the phospholipids (Figure 1F). In cholesterol esters of the liver tissue, a proportion of linoleic acid of less than one percent is found in all tested individuals (data not shown). In plasma of the cows, no significant differences can be found either in the proportions of omega −3 and omega-6 fatty acids or in the ratio between oleic acid and linoleic acid, so that an increased proportion of omega-3 and omega-6 fatty acids can only be observed in the liver tissue in the fraction of free fatty acids and in the triacylglycerides in diparous cows (Supplementary Figure 1).

### 3.2 Higher proportion of arachidonic acid in triacylglycerides of diparous cows *postpartum*

**FIGURE 2:**
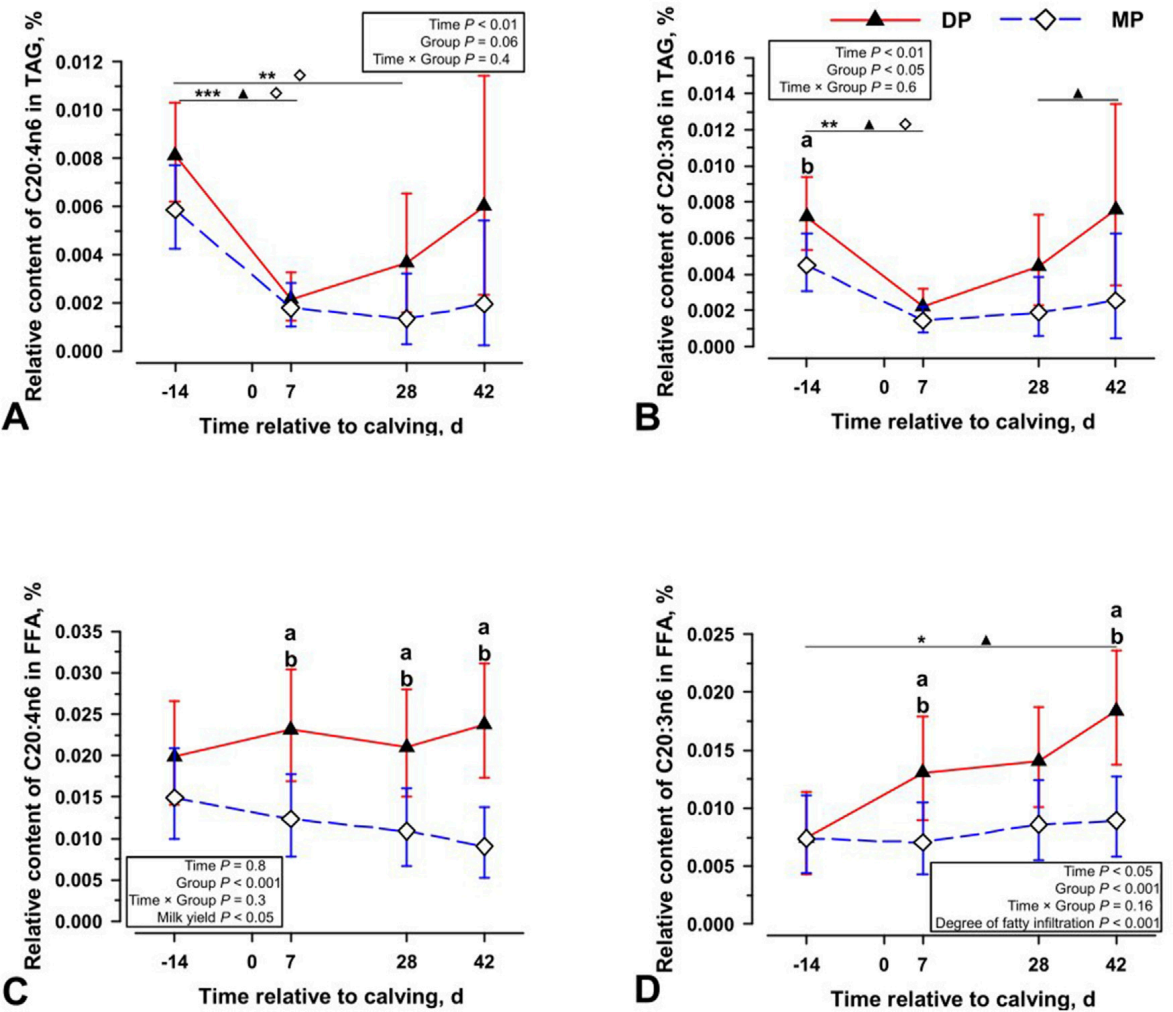
The relative content of C20:4n6 in triacylglycerol [TAG; **(A)**], relative content C20:3n6 in TAG **(B)**, relative content of C20:4n6 in free fatty acids [FFA; **(C)**], and relative content of C20:3n6 in FFA **(D)** at four periparturient sampling days [−14 = d 14 antepartum (a.p.); 0 = parturition; 7, 28, 42 = d 7, d 28, d 42 *postpartum* (p.p.)] in two groups (DP = lactation 2, n = 6; MP = lactation 3 or greater, n = 6) of German Holstein cows as Least Square Means (LSM) and their 95% confidence intervals (error bars). Differences between the time of sampling are marked with asterisks (*p < 0.05; **p < 0.01; ***p < 0.001), differences between time of sampling within the groups marked with symbols (▲ DP; ⍚ MP) and differences among the lactation groups at the respective time point marked with different lowercase letters: (a, b) indicate significant differences (*p* < 0.05).

After polyunsaturated fatty acids were observed to increase in the triacylglycerides of the liver tissue of diparous cows 42 days *postpartum*, the occurrence of inflammation-associated arachidonic acid (C20:4n6) and the related dihomo-gamma-linolenic acid (C20:3n6) in the bioptates was investigated. In both groups a significant drop in both PUFA can be detected between 14 days a.p. and 7 days p.p. in the fraction of triacylgycerides of liver tissue. (Figures 2A, B). In parallel to the occurrence of omega-3 and omega-6 fatty acids, an increase in both PUFA can be detected after day 7 p.p. in the liver tissue of diparous cows in the free fatty acids (Figures 2C, D). Both PUFA are present in the cholesterol ester fraction at levels well below one percent and were therefore not included in the analysis. Differences between the groups are neither observed in the phospholipid fraction nor in plasma (Supplementary Figure 2). Thus, only the fractions of triacylglycerides and free fatty acids from DP cows show a significant increase in arachidonic acid and dihomo-gamma-linolenic acid in liver tissue after parturition/calving.

### 3.3 Multiparous cows *postpartum* with elevated C16:1n7 concentrations

**FIGURE 3:**
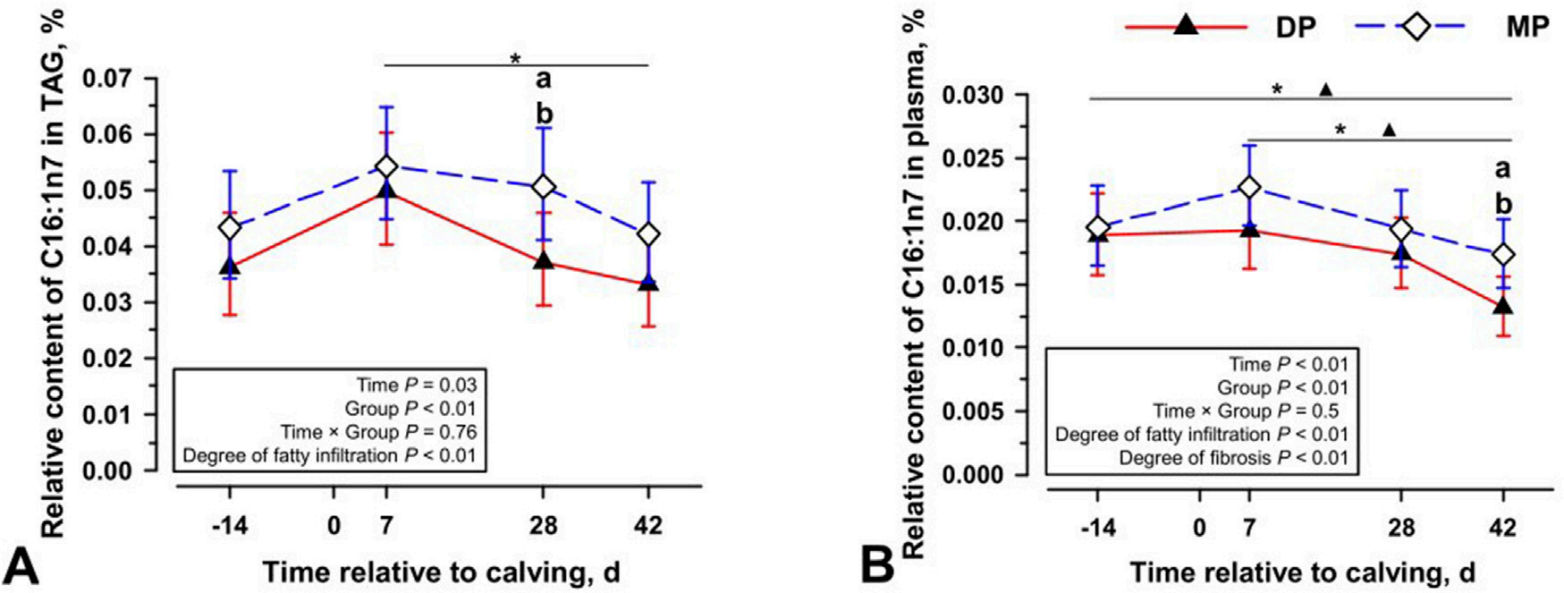
The relative content of C16:1n7 in triacylglycerol [TAG; **(A)**], and relative content C16:1n7 in plasma **(B)** at four periparturient sampling days [−14 = d 14 antepartum (a.p.); 0 = parturition; 7, 28, 42 = d 7, d 28, d 42 *postpartum* (p.p.)] in two groups (DP = lactation 2, n = 6; MP = lactation 3 or greater, n = 6) of German Holstein cows as Least Square Means (LSM) and their 95% confidence intervals (error bars). Differences between the time of sampling are marked with asterisks (*p < 0.05; **p < 0.01; ***p < 0.001), differences between time of sampling within the groups marked with symbols (▲ DP; ⍚ MP) and differences among the lactation groups at the respective time point marked with different lowercase letters: (a, b) indicate significant differences (*p* < 0.05).

**FIGURE 4:**
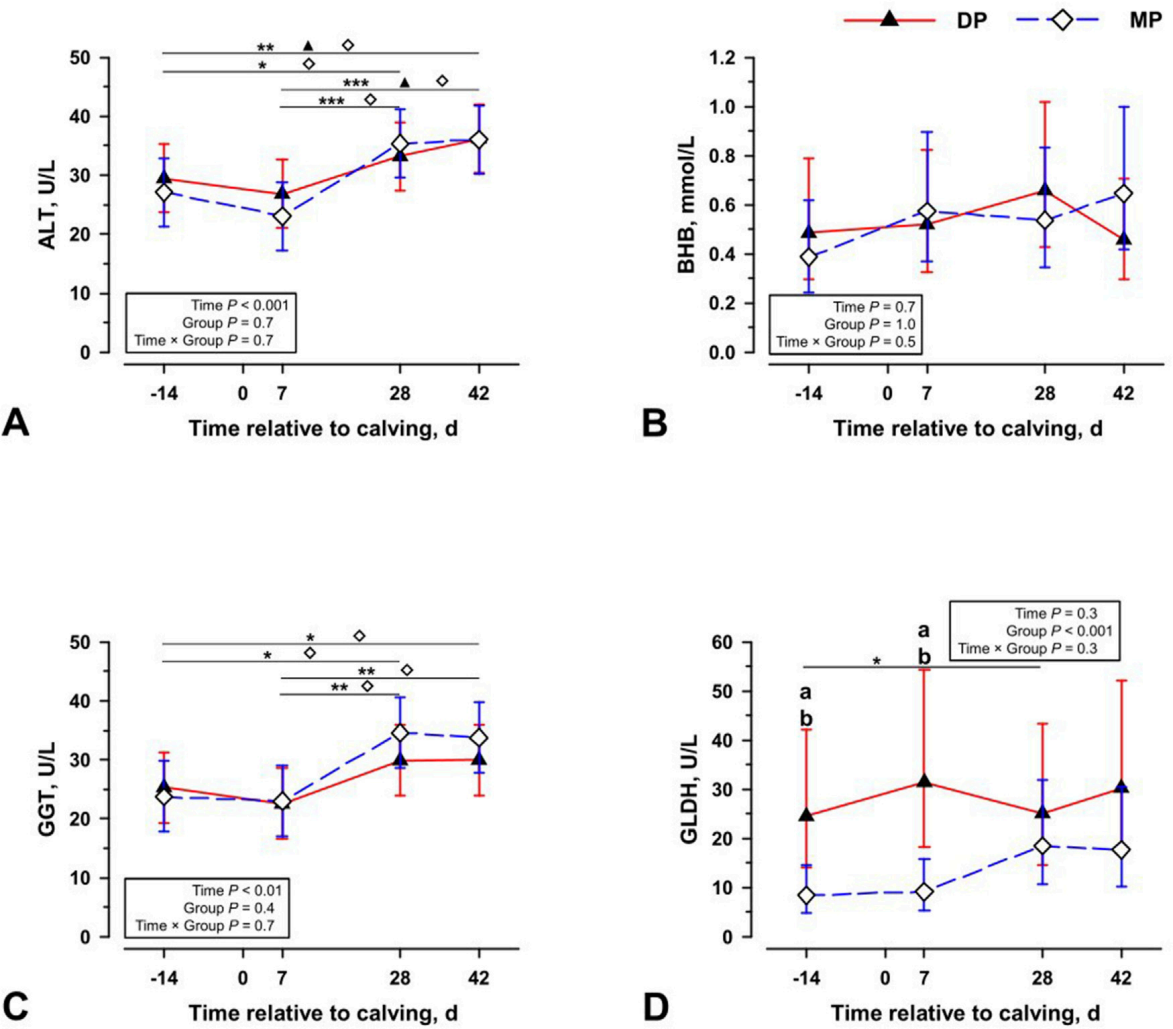
The plasma concentration of alanine aminotransferase [ALAT; **(A)**], beta hydroxybutyrate [BHB; **(B)**], γ-glutamyl transferase [GGT; **(C)**], and glutamate dehydrogenase [GLDH; **(D)**] at four periparturient sampling days [−14 = d 14 antepartum (a.p.); 0 = parturition; 7, 28, 42 = d 7, d 28, d 42 *postpartum* (p.p.)] in two groups (DP = lactation 2, n = 6; MP = lactation 3 or greater, n = 6) of German Holstein cows as Least Square Means (LSM) and their 95% confidence intervals (error bars). Differences between the time of sampling are marked with asterisks (*p < 0.05; **p < 0.01; ***p < 0.001), differences between time of sampling within the groups marked with symbols (▲ DP; ⍚ MP) and differences among the lactation groups at the respective time point marked with different lowercase letters: (a, b) indicate significant differences (*p* < 0.05).

Besides PUFA, only palmitoleic acid (C16:1n7) shows observable tendencies that reveal differences in the lipid metabolism of cows. 42 days after parturition/calving, increased amounts of palmitoleic acid are present in liver storage fats of diparous and multiparous cows (Figure 3A). However, the changes show different kinetics compared to those of PUFA. Figure 3A shows that before parturition/calving all cows have a similar content of C16:1n7 in the triacylglycerides and all other fractions of the liver tissue (Figures 3A, B; Supplementary Figures 3B, C). After parturition/calving, there is a considerable increase of approx. 70% in the triacylglycerides of the entire population. After 42 days, the C16:1n7 content falls back to the level before parturition/calving, (Figure 3A). In the fraction of PL of the liver tissue, a highly significant difference between both groups emerges at the last measured time point (Supplementary Figure 3B). A similar difference occurs in the plasma of the animals that reveals a distinctly lower drop in the C16:1n7 concentration in multiparous animals (Figure 3B). This change is not generally reflected in n7 fatty acids (Supplementary Figure 3A). After parturition/calving, the liver tissue and plasma of multiparous cows show a much lower drop in the C16:1n7 concentration. These changes can be observed without any apparent signs of adverse liver markers, such as increases in plasma alanine aminotransferase (ALT) levels. The ALT and GGT concentration (Figures 4A, C) changed over time, with higher concentrations on 28 days and 42 days p.p., but without significant differences in DP and MP cows. Also, the BHB concentration did not differ significantly between the groups and among the sampling days (Figure 4B). Overall, DP cows had significantly higher GLDH blood concentrations, but without significant changes in concentrations over the periparturient period (Figure 4D). Therefore, differences occurring in the palmitoleic acid concentration were not reflected in changes of traditional adverse liver markers.

### 3.4 Cell degeneration in multiparous cows *postpartum*

**FIGURE 5:**
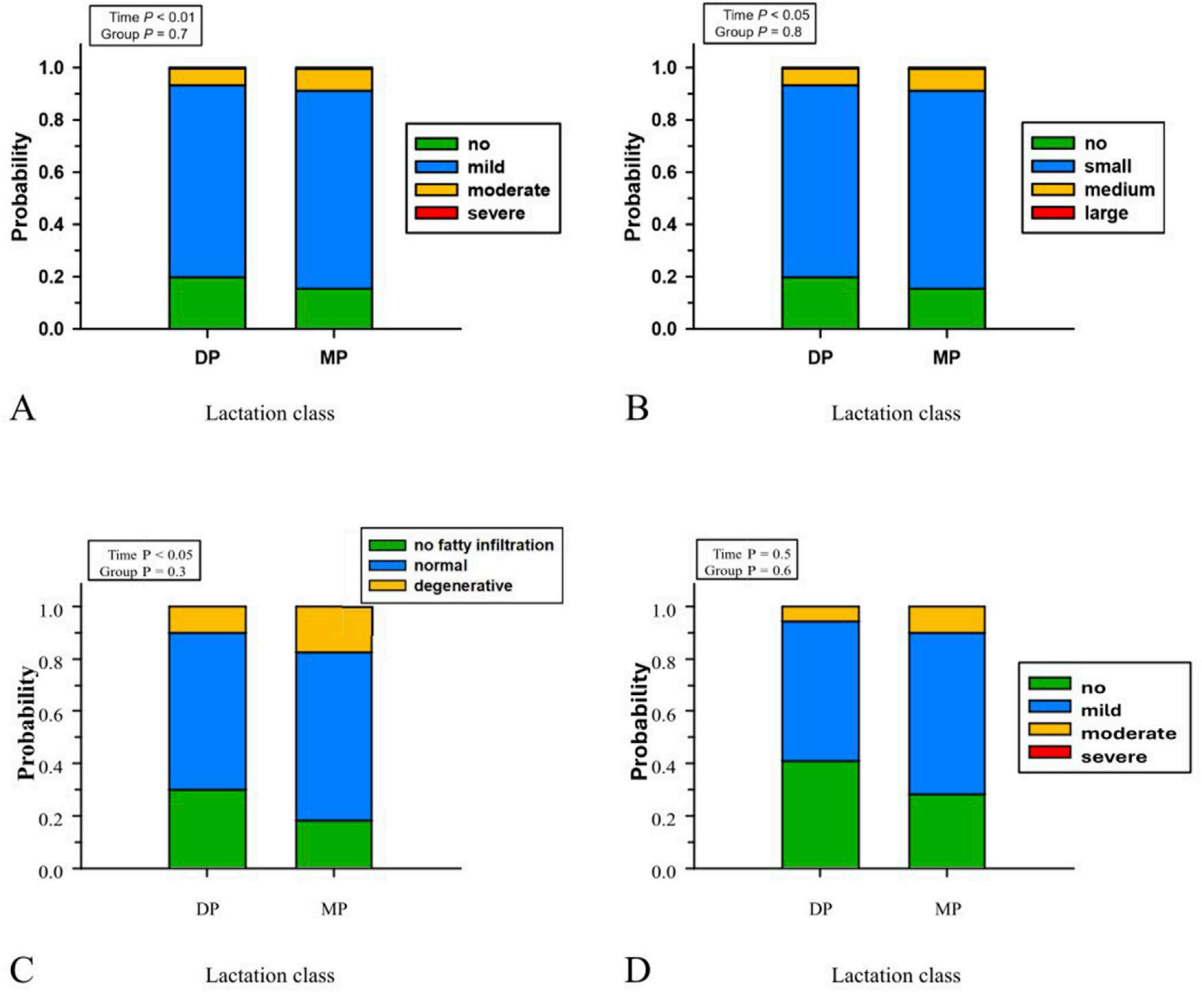
The rating of the degree of fatty liver infiltration **(A)**, size of lipid droplets **(B)**, hepatocyte degeneration **(C)**, and degree of perisinusoidal fibrosis **(D)** at periparturient sampling days [−14 = d 14 antepartum (a.p.); 0 = parturition; 7, 28, 42 = d 7, d 28, d 42 *postpartum* (p.p.)] in two groups (DP = lactation 2, n = 6; MP = lactation 3 or greater, n = 6) of German Holstein cows as probability estimation. **(A)**, level of infiltration/storage: no, mild, moderate, and severe infiltration/storage; **(B)**, size of lipid droplets: no, small, medium, large droplets; **(C)**, hepatocyte degeneration: no fatty infiltration, normal, degenerative; **(D)**, degree of fibrosis: no, mild, moderate, and severe fibrosis.

**FIGURE 6:**
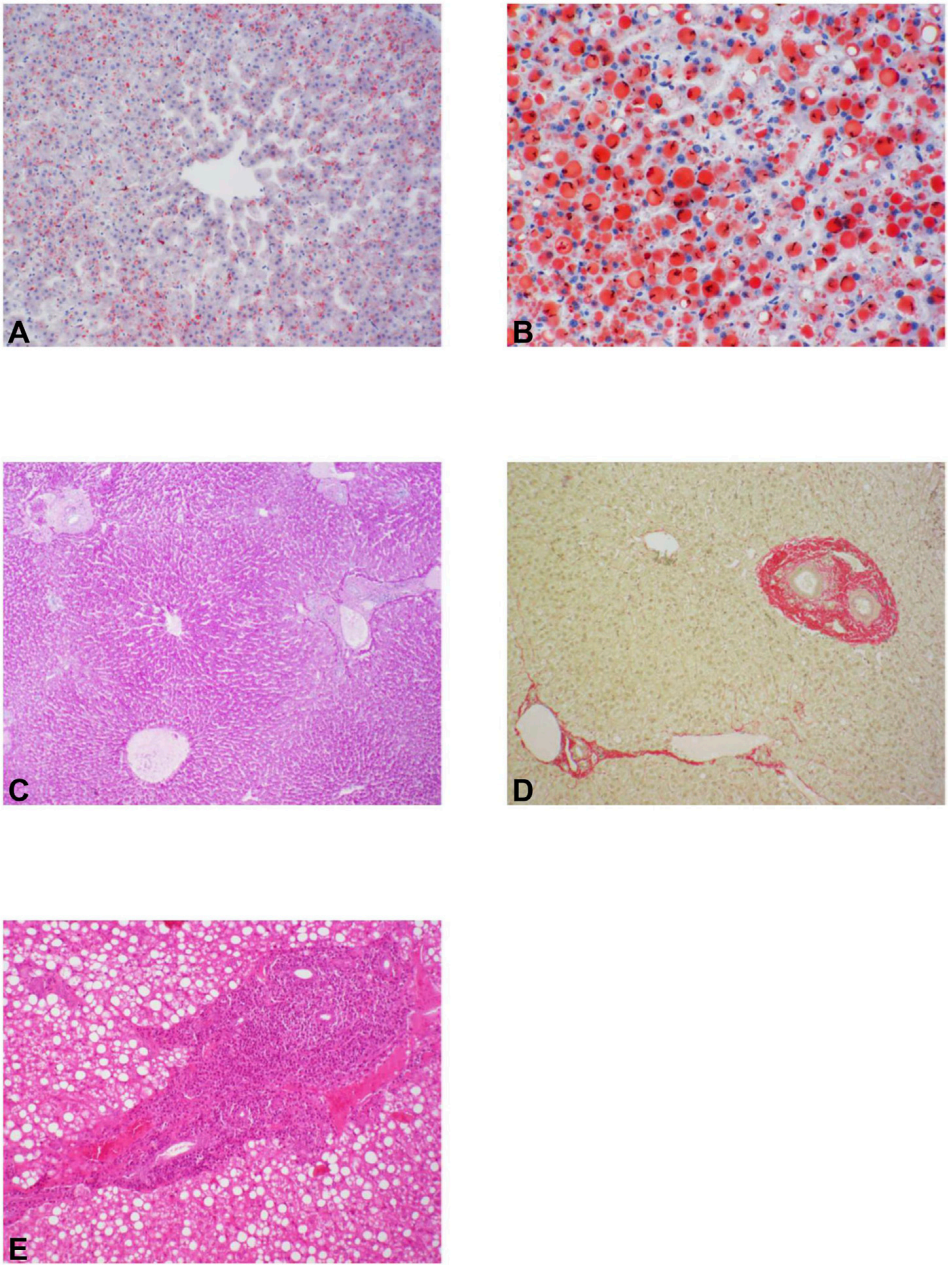
Degree of fatty infiltration of the liver in sections stained with Sudan III-hematoxylin: **(A)** moderate fatty infiltration, and **(B)** severe fatty infiltration. Degree of glycogen storage in sections with periodic acid-Schiff reaction: **(C)** moderate glycogen storage. Degree of overall fibrosis in sections stained with picrosirius red stain: **(D)** moderate fibrosis with collagen fiber formation around the central vein and portal vessels and collagen bridge formation between the individual portal fields. Assessment of the degree of hepatitis in hemalum-eosin sections: **(E)** severe hepatitis with bile duct proliferation. Sections are presented as examples.

During the transition period, the histological appearance of fatty infiltration, size of lipid droplets, and hepatocyte degeneration changed in hepatic tissue of the studied cows. The lactation number of the cows did not affect the degree of fatty liver infiltration, size of lipid droplets, hepatocyte degeneration, glycogen storage or incidence of perisinusoidal fibrosis. The degree of fatty infiltration of the hepatic tissue of all studied cows (N = 12) was mild (81%) to minimal (17%) at −14 days a. p. and increased thereafter. It was most severe at 7 days p.p. with odds of a lower degree of fatty infiltration with 31% mild and 58% moderate (Figure 6A). After calving from 7 days p.p. on, fatty infiltration decreased with increasing days in milk. The dynamic changes in the distribution of the size of lipid droplets in the liver were similar to the fatty infiltration; lipid droplets were scant (83%) and mostly small (16%) at −14 days ap (*p* < 0.01) but considerably larger at 7 days (73% small, 19% medium), 28 days (74% small, 17% medium), and 42 days p. p. (73% small, 19% medium), and lipid droplets were seen in almost all p. p. tissue sections. The odds of a lower degree of fatty infiltration in DP (Figure 5A) were 1.3 times greater than in MP. The odds of a lower lipid droplets sizes (Figure 5B) were 1.3 times greater in DP (0% severe, 6% moderate, 74% mild, 20% no fatty infiltration), compared to MP (1% severe, 8% moderate, 76% mild, 15% no fatty infiltration; Figure 6B). Only the degree of liver cell degeneration was affected with time. Degenerated cell nuclei in hepatocytes were rare (1%) at −14 days ap but occurred in 26%, 22%, and 28% of hepatocytes at 7 days, 28 days, and 42 days pp, respectively. Thereby, the odds of normal hepatocyte nuclei in DP cows were 1.9 times greater than in MP cows (Figure 5C). The degree of perisinusoidal fibrosis was neither affected by time nor group (Figures 6C–E). The odds of low-grade perisinusoidal fibrosis in DP (6% moderate, 53% mild, 41% absent) were 1.8 times the greater than in MP (10% moderate, 62% mild, 28% absent; Figure 5D). Therefore, increased cell degeneration and fibrosis are observed in MP cows p. p.

## 4 Discussion

In the *postpartum* dairy cows undergo significant metabolic stress due to the lipid mobilization triggered by the energy deficit. An altered lipid composition mainly of the liver reflects this stress (Bobe et al., 2004). The most notable difference is the consistent rise in the palmitoleic acid (C16:1n7) level across all cows around the time of calving (Figure 3). Studies demonstrate the role of fatty acid accumulation in hepatocytes, oxidative stress and mitochondrial dysfunction as predisposing factors for the development of NASH and hepatocellular carcinoma (HCC) (Gutiérrez-Cuevas et al., 2022). Research findings show a possible link between C16:1n7 and the progression of NASH to HCC. C16:1n7 was identified as a potential biomarker for the severity of NASH (Yoo et al., 2017). The serum concentration of the C16:1n7/C16:0 ratio was reported to be a potential diagnostic marker for non-alcoholic steatohepatitis (NASH) and showed significant correlations with inflammation, ballooning and fibrosis in liver histology (Yamada et al., 2019). In addition, the C16:1n7/C16:0 ratio was consistently found to correlate with the histologic presence of NASH (Bischoff et al., 2022). An increased C16:1n7/C16:0 ratio has also been associated with the progression of inflammation and fibrosis in NASH patients (Inada et al., 2019). Elevated levels of C16:1n7 are thought to play a role in intracellular signalling and endocrine communication, impacting lipid metabolism and insulin sensitivity while reducing Stearoyl-CoA Desaturase 1 (SCD-1) expression (Cao et al., 2008). Reduction of SCD-1 may lead to a decrease in monounsaturated fatty acid phosphatidylcholine (MUFA-PC), which is associated with hepatocyte proliferation and accumulation in HCC (Hall et al., 2021). C16:1n7 could perform a key function in the development of NASH and tumorigenesis in the liver and possibly indicate underlying changes as an early marker molecule.

The initial increase in C16:1n7 across all lipid fractions (Figure 3) from 12 days antepartum (a.p.) to 7 days *postpartum* (p.p.) in both groups suggests ongoing processes linked to metabolic liver stress. Subsequent decreases until 42 days *postpartum* may indicate a reduction in metabolic stress. Histological findings corroborate these findings, showing minimal fatty infiltrations in liver tissue 12 days a.p., peaking at 7 days p.p. and gradually decreased thereafter until the end of study, albeit not returning to initial levels (Pietsch et al., 2021). Analysis of total lipid concentration and lipid fraction yield in liver tissue supports these observations (Theinert et al., 2022). Noteworthy is the distinct difference of relative content of C16:1n7 between DP and MP cows at the final timepoint. While the initial time points show marginal differences, at 42 days p. p., DP cows approach the initial levels observed 12 days a. p., whereas MP cows exhibit consistently higher levels of C16:1n7 until the end of study.

The observed discrepancy could indicate impaired regeneration or enhanced degenerative processes in the livers of older cows, which may be related to the progression of NAFLD/NASH. Previous studies have identified evidence of a significantly higher incidence of severe perisinusoidal fibrosis in MP cows (Pietsch et al., 2021). Fibrotic tissue appears to correlate positively with the degeneration of hepatocytes. However, among animals analysed in this study there are no clear biochemical signs of increased cell death in the hepatocyte population (Figure 4). The histological trends, however, indicate elevated hepatocyte degeneration and perisinusoidal fibrosis in MP cows liver tissue (Figure 5). This suggests that the observed changes in C16:1n7 are an early indicator of cell stress and possible changes in liver tissue. Future studies will have to observe when cell degeneration starts.

At the same time, the only other changes that could be observed in the cows examined concerned PUFA. Higher concentrations of omega-3 and omega-6 fatty acids, arachidonic acid (C20:4n6) and dihomo-gamma-linolenic acid (C20:3n6) were identified in the free fatty acids and especially in the storage fat of the liver tissue in young cows (Figures 1, 2). The latter PUFAs can be enzymatically converted into proinflammatory eicosanoids by Cyclooxygenase-2 (COX-2), identified as markers for inflammatory processes in NAFLD and higher in HCC (Yang et al., 2011; Sztolsztener et al., 2020). However, the accumulation of these fatty acids in storage fat suggests against their contribution to the inflammatory process. In fact, the two PUFAs behave kinetically and in the same manner as the substrates of their syntheses, the omega-3 and omega-6 fatty acids. In contrast, the kinetics of C16:1n7 are completely different. In addition, an increased level of C16:1n7 is not only found in the free fatty acids and the storage fat, but in all lipid fractions of the liver tissue and even in the plasma. These differences appear to indicate different synthesis activities. While C16:1n7 is increasingly found throughout the system and is therefore probably synthesized, the PUFA are only found in the storage lipid of the liver and are likely recruited from the periphery. Consequently, the differences in concentrations of omega-3 and omega-6 fatty acids, C20:4n6 and C20:3n6 indicate a different content of fatty acids in the subcutaneous fat storage and the increased occurrence of arachidonic acid could merely be a consequence of the increased mobilization of the synthesis substrates. A greater temporal proximity of the grazing of young cows to lipid mobilization is consistent with these results. The differences in the recruitment of PUFA appear to be independent of the increased occurrence of C16:1n7 and the persistently higher concentration of this fatty acid, especially in old cattle. Therefore, only the analysis of C16:1n7 has the potential to identify early liver changes that might be observed in the progression of NAFLD/NASH.

It should be noted that the sample size of our study limits the interpretation of the results. However, the selected animals showed similar and significant changes in production level (BCS, milk yield), blood chemistry traits (NEFA, BHB) and liver fat content as part of normal periparturient changes (Theinert et al., 2022). The differences between diparous and multiparous cows are clear and correspond with the results of other studies (Benedet et al., 2019; Djokovic et al., 2019; Takahashi et al., 2021). Based on the clinical findings, the selected cows can be considered a homogeneous cohort. HCC is the most frequently (84.2%) observed hepatic neoplasm in cattle, and cows are more frequently affected than males because they reach an age at which the probability of HCC increases (Vielmo et al., 2020). The probability of a lower degree of fatty infiltration was the same (OR 1.3-fold) in lactation classes 2 and 3 (0% and 0% severe, 1% and 1% moderate, 71% and 77% mild, 22% and 17% no fatty infiltration). The degree of fatty infiltration changes over time, and the odds of less fatty infiltration at day −14 were 91.8 times higher than at day 7 pp, 46.6 times higher at day 28 pp and 21.6 times higher at day 42 pp. Despite the fatty infiltration, the cows were examined as clinically healthy. Our study consequently identifies molecular changes that may precede NASH and HCC development. Therefore, the model of lipid mobilization in the dairy cow provides the opportunity to investigate the molecular basis of lipid associated liver disease development.

## 5 Conclusion

Differences in lipid metabolism in cows after calving may be a natural model for the metabolic changes associated with NAFLD/NASH and tumor development in the liver. Especially since kinetic observation of reversible recruitment of storage fat and its impact cell stress signaling in the living organism is feasable. We observed differences in the concentration of PUFA in liver storage fat, presumably due to different proportions of these fatty acids in subcutaneous fat storage, as well as changes in palmitoleic acid (C16:1n7), suggesting that C16:1n7 increases with metabolic stress and may indicate greater changes in liver tissue in multiparous cows. The increase in C16:1n7 levels occurred in healthy cows and without established markers indicating cell degeneration, perhaps indicating early signs of metabolic stress of the liver.

## Data Availability

The original contributions presented in the study are included in the article/Supplementary Material, further inquiries can be directed to the corresponding author.
