# Supplementary figures and images for: A potential gateway to understanding liver disease development: peripartum lipid fluctuations in dairy cows
Source: Front Cell Dev Biol. 2024 Nov 26;12:1370717. doi: 10.3389/fcell.2024.1370717 (PMC11628505; doi:10.3389/fcell.2024.1370717)

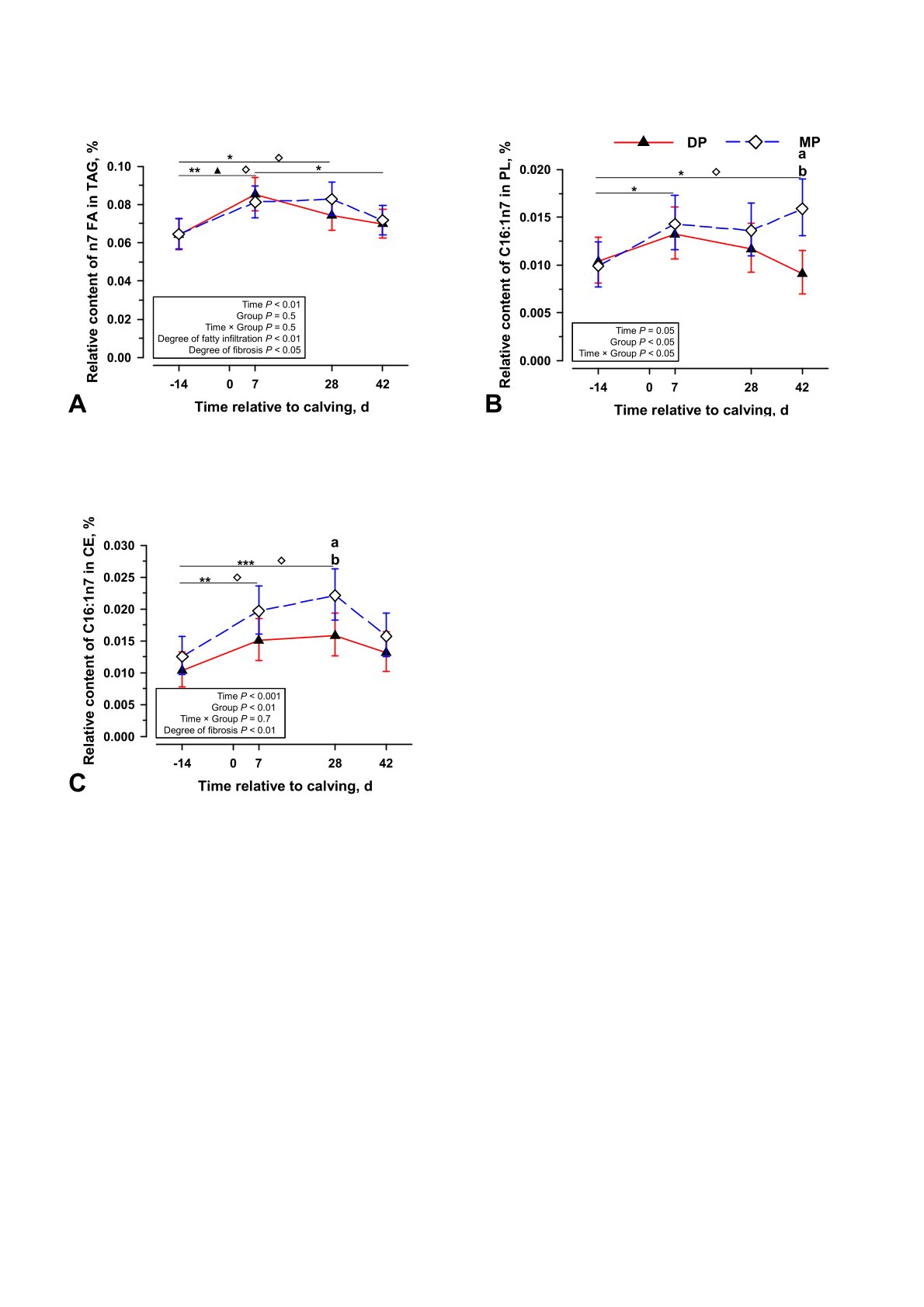

Supplement: Supplementary file 1 [file Image3.jpeg]

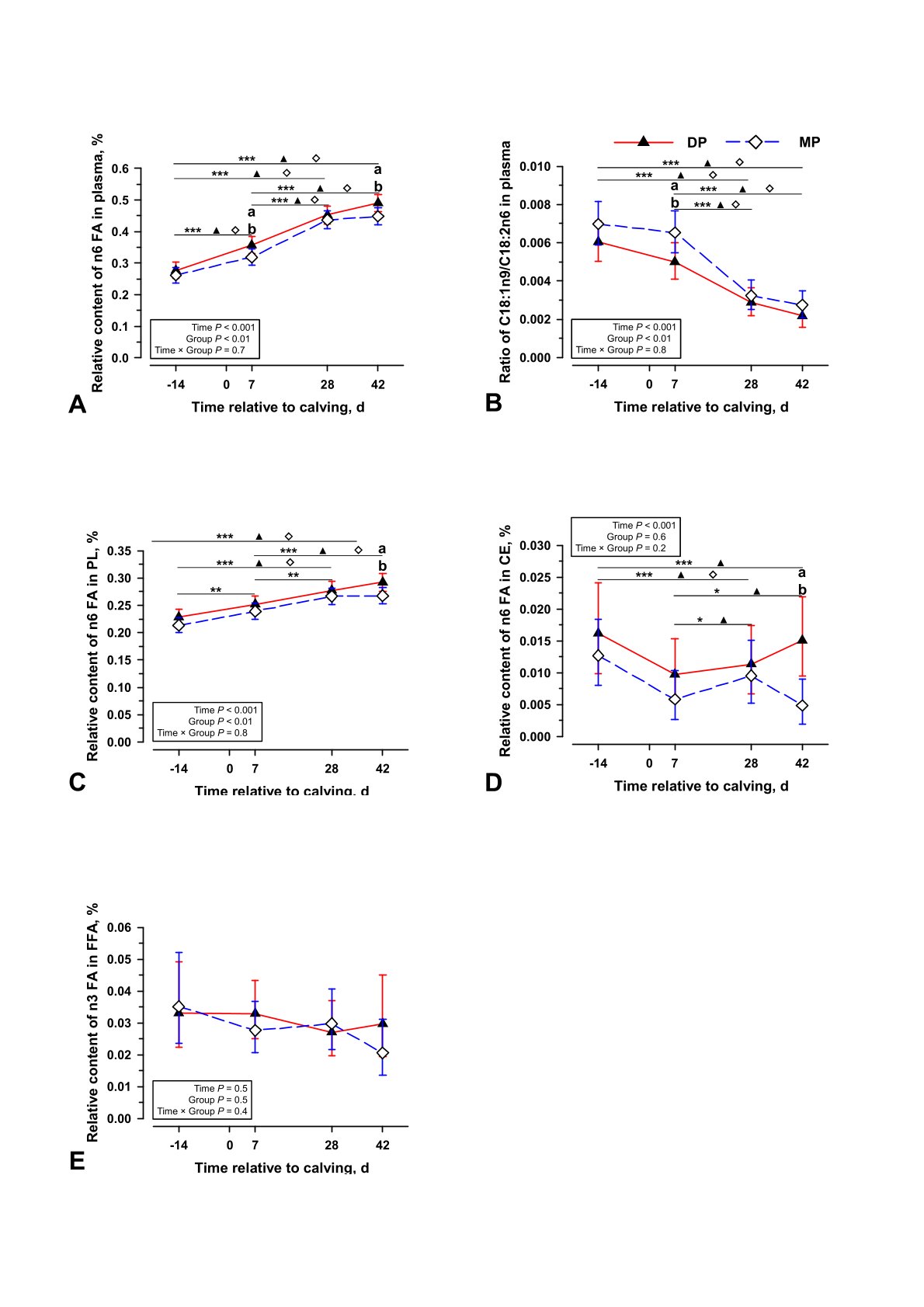

Supplement: Supplementary file 3 [file Image1.jpeg]

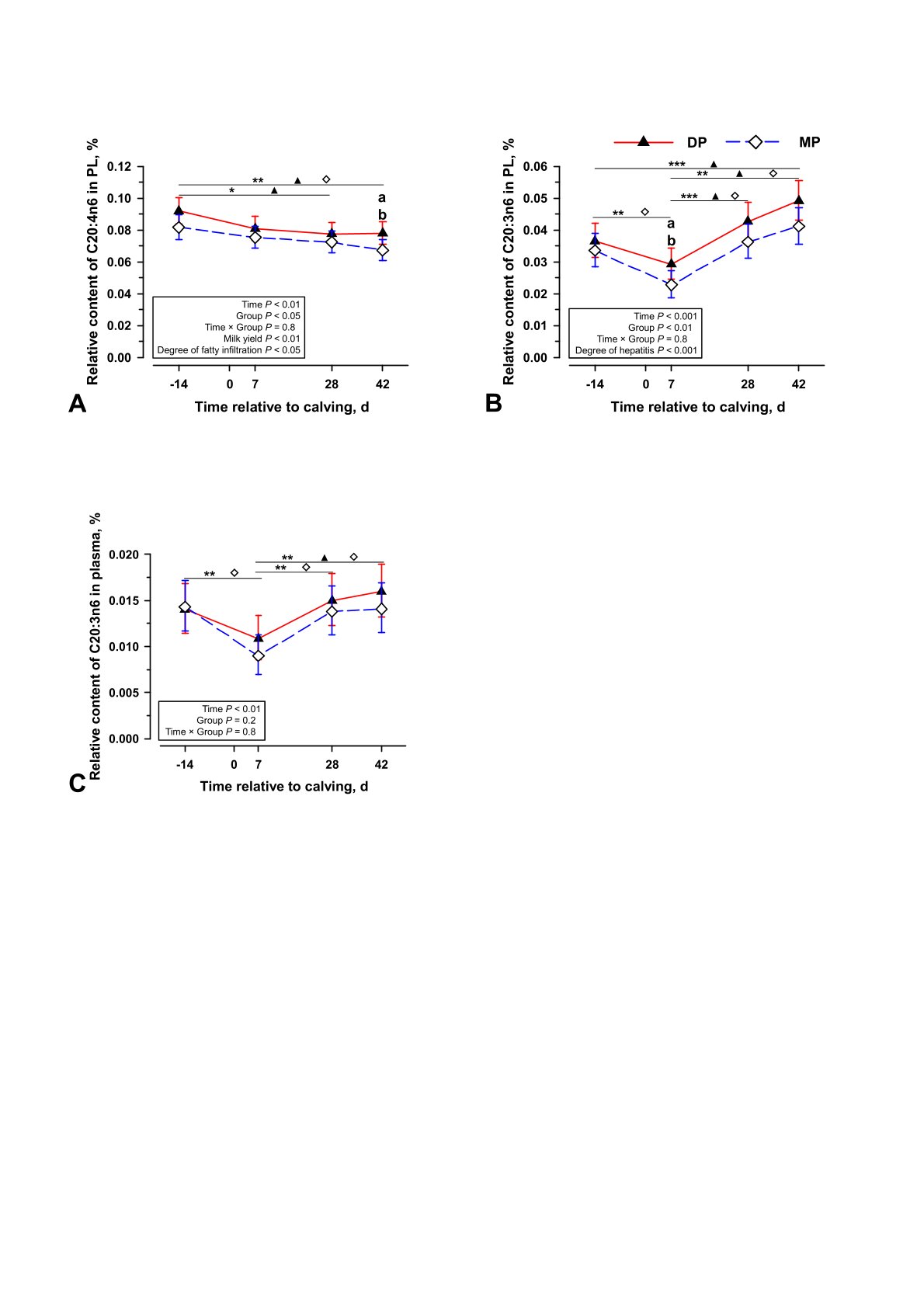

Supplement: Supplementary file 4 [file Image2.jpeg]
