# Supplementary material for: A potential gateway to understanding liver disease development: peripartum lipid fluctuations in dairy cows
Source: Front Cell Dev Biol. 2024 Nov 26;12:1370717. doi: 10.3389/fcell.2024.1370717 (PMC11628505; doi:10.3389/fcell.2024.1370717)
Supplement: Supplementary file 2 [file Table1.docx]

| **ID** | **Milk yield**  **(kg, 305-d production in previous lactation)** | **Birth date**  **(Age in years)** | **Calving date** | **Lactation** |
| --- | --- | --- | --- | --- |
| DE14040 83898 | 8865 | 28.03.2012 (4) | 05.12.2015 | 2 |
| DE14040 32709 | 14470 | 16.08.2011 (4) | 06.12.2015 | 3 |
| DE14039 31072 | 10413 | 10.05.2010 (6) | 08.12.2015 | 4 |
| DE14040 32632 | 12508 | 09.07.2011 (4) | 12.12.2015 | 3 |
| DE14042 70606 | 9946 | 31.07.2012 (4) | 02.02.2016 | 2 |
| DE14043 58570 | 10436 | 23.08.2012 (3) | 30.01.2016 | 2 |
| DE14042 70267 | 11766 | 06.02.2012 (4) | 05.02.2016 | 3 |
| DE14043 58510 | 7685 | 27.06.2012 (4) | 20.04.2016 | 2 |
| DE14034 02263 | 10147 | 27.10.2008 (7) | 23.04.2016 | 6 |
| DE14046 21515 | 8870 | 23.07.2013 (3) | 28.07.2016 | 2 |
| DE14046 21527 | 11818 | 01.08.2013 (3) | 28.8.2016 | 2 |
| DE14040 83587 | 11505 | 09.06.2011 (5) | 9.9.2016 | 4 |

**Supplementary Table I**

**Supplementary Table II**

| **lactation group** | **time** | **Mean value of triacylglycerol in % per g liver fresh weight** | **Mean value of cholesteryl esters in % per g liver fresh weight** |
| --- | --- | --- | --- |
| DP | -14 | 0,31 | 0,10 |
| MP | -14 | 0,37 | 0,13 |
|  |  |  |  |
| DP | 7 | 2,12 | 0,18 |
| MP | 7 | 2,72 | 0,24 |
|  |  |  |  |
| DP | 28 | 1,89 | 0,18 |
| MP | 28 | 3,23 | 0,37 |
|  |  |  |  |
| DP | 42 | 0,75 | 0,17 |
| MP | 42 | 1,45 | 0,21 |
